# Comment on “The Effects of Bariatric Surgery Weight Loss on Knee Pain in Patients with Osteoarthritis of the Knee”

**DOI:** 10.1155/2013/517803

**Published:** 2013-04-24

**Authors:** Janice Lin, Manish Parikh, Jonathan Samuels

**Affiliations:** NYU Langone Medical Center, New York, NY 10016, USA

It was with great interest that we read “The Effects of Bariatric Surgery Weight Loss on Knee Pain in Patients with Osteoarthritis of the Knee” by Edwards et al. [1]. As other studies have shown, obesity is an incrementally modifiable risk factor for the development and progression of knee osteoarthritis (KOA) [2, 3]. Any opportunity to treat obesity and potentially limit KOA progression and disability will be an important public health strategy. Bariatric surgery is superior to regimented dietary and exercise programs in helping obese patients achieve and maintain weight loss [4, 5], and thus we think the authors focused on an important potential option to help obese patients avoid total joint replacement surgery.

We found it valuable that the authors conducted a joint-driven and hypothesis-driven study to track pain and functional improvement in patients who underwent the three types of bariatric surgery: gastric bypass, sleeve gastrectomy, and laparoscopic adjustable gastric banding (LAGB). We agree with the authors that the existing literature is limited in examining the effect of bariatric surgery on KOA, particularly with confirmation of patients' radiographic KOA and the utilization of validated tools such as Western Ontario and McMaster Universities (WOMAC) Index of Osteoarthritis, Knee Osteoarthritis Outcome Score (KOOS). 

 The authors provided encouraging data, but it was unclear whether medications including acetaminophen, nonsteroidal anti-inflammatory drugs, tramadol, narcotics, topical agents, or intra-articular injections were given to patients for their knee pain during the study period. Any change in a patient's treatment regimen for KOA may contribute to the improvement of pain and function, beyond weight loss alone from bariatric surgery, and impact the findings. While it is unreasonable to ask patients to avoid any pain medication during such studies, it would be helpful to the readers to view the treatments and draw their own conclusions.

 In addition, the study does not report the individual ages (ranging from 18 to 70 years), demographics, or body mass indices (BMIs) of the 24 patients enrolled, or, perhaps, mean ages and BMIs of the 3 surgical subgroups. In our center we are seeing a subcohort of often younger patients with painful knee OA but only mild-moderate obesity (BMI 30–35) who would qualify for the less-invasive LAGB by recent FDA guidelines [6], but insurance companies are often unwilling to pay for the surgery. Thus, we are currently piloting a prospective study for patients with mild-moderate obesity (BMI 30–35) and severe pain from KOA, evaluating the effectiveness of LAGB that has shown retrospective promise for such knee pain with minimal risks [7–9]. 

 In conclusion, this study supports recent arguments [10–12] that weight loss from bariatric surgery can provide a significant improvement in patient-reported KOA-related pain and disability. In addition to the mechanical load reduction, each of the three surgical methods may have distinct biochemical and metabolic consequences on knee OA—as other authors have begun to explore the role of inflammation and joint biomarkers from adipose tissue [13]. Such investigation will only further our understanding of the interplay between mechanical and cytokine-driven effects of obesity on knee pain and function.
